# Automatic Waypoint Generation to Improve Robot Navigation Through Narrow Spaces

**DOI:** 10.3390/s20010240

**Published:** 2019-12-31

**Authors:** Francisco-Angel Moreno, Javier Monroy, Jose-Raul Ruiz-Sarmiento, Cipriano Galindo, Javier Gonzalez-Jimenez

**Affiliations:** Machine Perception and Intelligent Robotics Group (MAPIR), Dept. of System Engineering and Automation Biomedical Research Institute of Malaga (IBIMA), University of Malaga, 29071 Málaga, Spain; jgmonroy@uma.es (J.M.); jotaraul@uma.es (J.-R.R.-S.); cga@uma.es (C.G.); javiergonzalez@uma.es (J.G.-J.)

**Keywords:** mobile robots, robot navigation, robot localization, robot deployment, waypoint generation

## Abstract

In domestic robotics, passing through narrow areas becomes critical for safe and effective robot navigation. Due to factors like sensor noise or miscalibration, even if the free space is sufficient for the robot to pass through, it may not see enough clearance to navigate, hence limiting its operational space. An approach to facing this is to insert waypoints strategically placed within the problematic areas in the map, which are considered by the robot planner when generating a trajectory and help to successfully traverse them. This is typically carried out by a human operator either by relying on their experience or by trial-and-error. In this paper, we present an automatic procedure to perform this task that: (i) detects problematic areas in the map and (ii) generates a set of auxiliary navigation waypoints from which more suitable trajectories can be generated by the robot planner. Our proposal, fully compatible with the robotic operating system (ROS), has been successfully applied to robots deployed in different houses within the H2020 MoveCare project. Moreover, we have performed extensive simulations with four state-of-the-art robots operating within real maps. The results reveal significant improvements in the number of successful navigations for the evaluated scenarios, demonstrating its efficacy in realistic situations.

## 1. Introduction

Assistive robots are expected to play an important role in our daily lives. In the last decade, we have witnessed a considerable boost in this topic, with the development of new capabilities and skills for the robot that allows it, for example, to act as in-home caregivers capable of feeding disabled people [1], to support independent locomotion with smart wheelchairs [2], to promote cognitive activities for elders [3,4], to assist the user in finding lost objects [5] or unnoticed gas leaks [6], or to provide entertainment and health-related social network interactions [7]. In Europe, the interest in assistive robotics is clearly revealed by the number of EU research projects funded in the very recent years (e.g., [8,9,10,11,12,13]).

Despite these advances, many barriers still remain in order to achieve fully autonomous and reliable robots working at homes. One of the practical hurdles to overcome is that of performing safely and robustly when negotiating tight spaces in houses, like corridors, corners or doorways. Ideally, provided that a feasible path exists, current path planners (as those available in the Robotics Operating System (ROS) [14]) are able to find proper trajectories to overcome these complicated navigation areas. But, according to our experience in real deployments, robots find serious problems for performing safely and following the planned path. The reasons for this undesirable behavior are diverse, including, among others, noise and miscalibration errors of the robot sensors, or inaccuracies in robot localization or in the motion execution.

**Figure 1:**
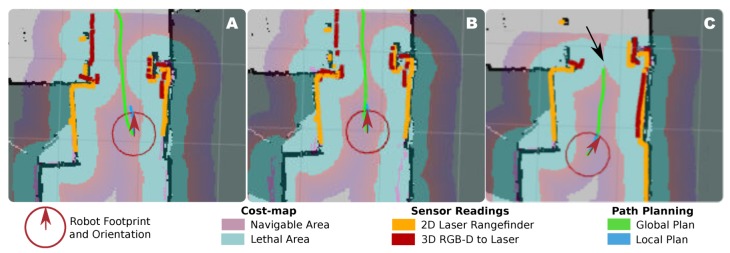
Illustration of three different situations (**A**–**C**) experienced by a mobile robot when traversing a door. The navigable area is, as perceived by the robot, the result of multiple factors like its localization, the obstacles detected by the on-board sensors, and their corresponding errors. The latter have an important impact in the surroundings of narrow areas (i.e., the door) as the robot may perceive there is not space for it to pass trough.

To illustrate this, Figure 1 shows the problem of autonomous navigation of a mobile robot when passing through a narrow area. This figure presents the cost-map [15] built by the robot and the sensor readings when facing this navigation task. In short, a cost-map represents the cost of traversing each position in the map according to certain criteria, which, in this case, is the distance to the detected obstacles. Specifically, we illustrate the scenario where a mobile robot (with a circular footprint of 52 cm ø) is commanded to pass through a standard-sized door (72 cm wide) connecting two rooms. Three different situations are displayed in this example: (A) the desired scenario, where the robot is facing the door from a close initial position and almost following a straight path, (B) a more challenging situation where the combination of the sensors’ noise, the calibration errors between the sensors (i.e., the 2D laser scanner and a 3D RGB-D camera, in the example), and the error in the robot localization reduce the navigable area in the door frame, yet, being feasible the estimation of a path, and (C) an illustration of a faulty navigation attempt where the robot perceives there is not space to pass through the door and, therefore, will abort the navigation. The critical issue is, in all cases, the relatively small margin of operation for the robot to plan and execute the navigation. Theoretically, the robot in our example has a margin of ∼20 cm to pass through the door but, as can be seen in the corresponding cost-maps, the robot sensors do not perceive such a clearance but a much more restricted one of just a few centimeters. This, in practice, causes the robot to fail its navigation on many occasions, limiting the accessibility to other rooms and spaces in the house and, consequently, its practical value as an assistant. It must be stressed that in this figure only errors related to noise, calibration and localization can be represented, yet, we still need to account for the inaccuracies of the reactive navigator when following the global plan, and the motion execution errors that may also affect the navigation result.

Seeking to improve the robustness and tolerance of navigation systems against the multiple sources of error that apply, in this work we present a practical solution to the problem of navigating through narrow areas that imposes the robot to traverse them by following a trajectory as straight as possible, while keeping into consideration the costs of navigating close to obstacles. Straight trajectories are especially favorable when dealing with narrow areas because they are simple to plan and execute due to the fact that they significantly reduce errors related to motion control, drift, and deviation from the optimal path. Additionally, this configuration also minimizes the impact of the radial-distortion error that typically presents all kinds of cameras in their measurements, as the obstacles to be considered (like, for instance, the door frames) fall in front of the robot.

More specifically, our approach involves two steps: on the one hand, the detection of the narrow areas along the navigation path of the robot that may lead to a poor navigation realization, and, on the other hand, the modification of the planned path to enforce a straight trajectory when passing through them. The former is attained by an algorithm that, taking into account the robot footprint, the occupancy grid map of the environment and the desired navigation path to reach the goal, automatically locates the cumbersome areas and defines, for each one, a single identification point, referred in this work as the critical navigation point (CNP). Then, in the second step, for each CNP we propose the unsupervised generation of a set of auxiliary navigation points (ANPs) that will be used as intermediate navigation waypoints, and that takes into consideration the characteristics of the local area around the narrow zone. Finally, a re-planning of the navigation path is performed, including the corresponding ANPs to reach the final goal.

In summary, this work provides a practical solution to the navigation of autonomous robots through narrow spaces that automatically fosters the robot to traverse such difficult areas following a straight path. Concretely, the contributions are twofold:

- The identification, without any human intervention, of cumbersome zones in the robot’s working area during an initial inspection stage (typically narrow zones such as passages or doors).
- The automatic and on-the-fly generation of a pair of auxiliary navigation waypoints for each cumbersome zone, which modify the robot trajectory and ensure proper navigation through such zones.

To validate our proposal, we first present experiments with simulated robots operating in real maps of typical houses. We consider in this experiment multiple footprints of state-of-the-art social robots. Navigation results when simulating all the possible trajectories in the maps are provided, evaluating the collisions and unsuccessful navigation attempts with and without our proposed automatic waypoint generation system. Finally, a real experiment has been performed with the Giraff.X social robot [16], used in the H2020 MoveCare project. We analyze the increase of the ratio of successful trajectories when applying our proposed method while also enabling a comparison with the simulated experiments.

In the following, we present in Section 2 a survey on the different strategies proposed in the robotics literature to deal with autonomous navigation in narrow spaces. Our proposal is thoroughly described in Section 3, including the algorithms developed for determining a successful robot trajectory. Section 4 and Section 5 present the experiments in both simulation and within a real environment, discussing the obtained results. The conclusions of the evaluation and future works are finally presented in Section 6.

## 2. Related Work

The navigation through narrow areas is a problem with an eminently practical component that is shared by most autonomous vehicles, including autonomous cars [17], mobile robots [18,19] or ships [20,21,22]. The challenge of this particular type of navigation lies in the little room available for maneuvering, leading, in many cases, to situations where the safety of the autonomous vehicle or the elements in the environment cannot be granted [23]. Besides, mainly due to the inherent inaccuracies in the vehicles’ sensory systems, as well as other sources of error related to localization, path planning and motion control, navigating through such narrow areas might be cumbersome or even impossible during normal operation, generating unreachable zones in the environment. In this work, we focus on mobile robotics systems that operate in real-world applications, particularly service robots in home environments. Of particular interest are the robotics systems whose size and dynamics make them potentially harmful, i.e., those that are susceptible to hurting someone or something if not properly controlled.

Among the different approaches proposed to deal with this challenging problem, multiple works have focused on the path planning component of the navigation, proposing specific algorithms to work under narrow areas. The task of path planning for nonholonomic systems is not trivial, being affected by the concurrent presence of geometric and kinematic constraints [24]. The latter have lead to the separation in two phases of the planning process, namely global, accounting for the global geometric constraints, and local, which takes into consideration the currently sensed data and the kinematic restrictions of the robot to follow the global path. Planners specifically designed to face narrow navigable areas include [17,25,26] for the case of car-like robots, proposing geometric path planners able to generate good quality paths with multiple maneuvers, or [27] where a novel two-stage path planner combining the efficient sampling Bridge-Test [28] algorithm for the identification of the critical regions (i.e., narrow areas) with rapidly-exploring random trees (RRTs) was reported for multi-d.o.f robot path planning problems. Following this approach, the work in [29] proposes a variation of the RRTs for balancing local and global information and achieve better results, especially in highly constrained environments. As another example, in [30], the authors presented a specific obstacle avoidance system to improve the navigation within narrow aisles of a warehouse using ultrasonic sensors.

In this context, the tasks of detecting and traversing doors have been largely studied by the robotics community as a particular example of a narrow area that robots should be able to navigate through. In [31] a 3D vision-based method for detecting doors was presented together with an adaptive controller to make the robot cross them perpendicularly. Similarly, a geometric approach based on confocal curves (i.e., hyperbolae, ellipses, and circles) for navigating a nonholonomic robot through a door by using only a monocular camera is presented in [32]. The work in [33] implements an identification system of the door aperture (e.g., closed, partially open, wide open, etc.) to allow the robot to judge if crossing the door was a suitable operation or not. In [34], in turn, the problem was addressed for the case of an autonomous wheelchair by proposing a dynamic path planning algorithm implementation based on successive frontier points determination [35]. Also in this context, the work in [36] proposes a low-cost system for electric wheelchair navigation in complex environments based on a pan-tilt camera and visual markers placed on the door frames. Our work generalizes this problem by not considering a particular scenario that imposes the presence of doors or corridors (therefore not needing to detect and identify specific features), but any narrow area in the environment where the robot should safely navigate through.

As another interesting approach, there exist several works that define a topology to assist robot navigation. In [37] doors are explicitly included in the topology and a detection algorithm of the door opening-state is presented to estimate the optimal path. Likewise, in [38], the environment is modeled as a topology that takes into account rooms, corridors and doorways. The nodes in the topology stand for places where a change in the navigational strategy occurs (behavior-based navigation), proposing to set nodes in front of each door and at each corridor crossing.

Other approaches include the installation of external cameras to guide the robots through narrow areas where only one at a time can navigate [39], or those less fancy, yet common, approaches based on the restriction of the navigation space in order to exclude areas with potential danger. Among others, environments with the presence of carpets and/or full-length mirrors are usually discarded as zones for proper navigation, as well as those with doorways or corridors too narrow (with respect to the width of the robot) to ensure a smooth navigation [40]. Naturally, the opposite solution has also been explored: to constrain the robot dimensions [41] or the motion design [42] to allow the robotic system to navigate through narrow areas, even for semi-autonomous vehicles where the control of the human operator can be overrode in case of danger of collision [43].

Our proposal can be categorized as a topology-like solution as it involves the definition of additional navigation waypoints (similar to the nodes in a topology). Yet, we handle each narrow area independently, that is, we do not build a connectivity graph to estimate the optimal path. Moreover, our solution generalizes to any narrow area in the environment without relying on the detection of specific features to identify doors or corridors, for example, hence becoming immune to recognition errors. Finally, our approach takes into consideration the local characteristics of the narrow areas (being characterized by a navigation cost) to optimize the position of the additional waypoints.

## 3. Navigation Assistant

Our proposal for a navigation assistant builds upon the detection of cumbersome areas and the automatic generation of auxiliary navigation points that the robot must traverse to arrive at certain difficult-to-reach destinations.

Thus, let us formally define a node as a destination point within the map that the robot should be able to reach autonomously, and a failure zone as the location in the map where the robot either experienced collisions or it was unable to find a proper trajectory to reach the destination. Typically, the metric map of the environment is built during an initial robot navigation that is manually driven by a human operator. Then, given the high amount of factors involved in the navigation (e.g., motor control, slippery floor, sensor noise, path planner configuration, etc.), we propose the detection of failure zones by commanding an initial exploratory mission that autonomously traverses all the possible trajectories between the nodes in the map, while looking for navigation problems.

**Figure 2:**
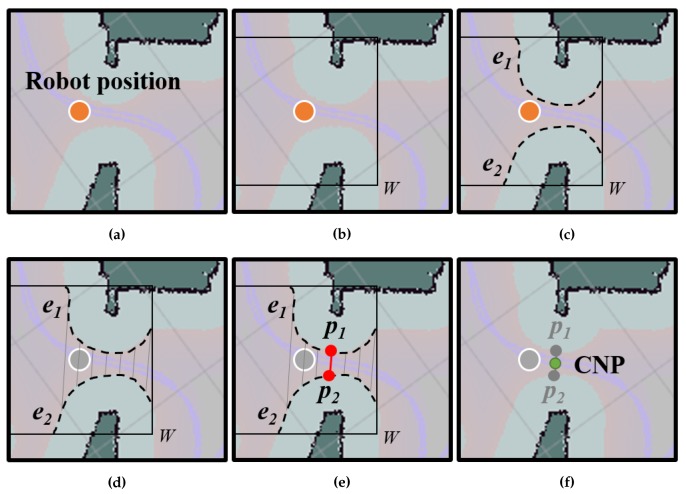
Detection of a critical navigation point (CNP) in a cost-map. In this case, the pink areas correspond to navigable space. (**a**) From the robot position at a failure zone (in orange), (**b**,**c**) we detect cost edges within a window W around it. (**d**) All the distances between edges $e_{1}$ and $e_{2}$ are computed and (**e**) the points yielding the minimum distance are selected ($p_{1}$ and $p_{1}$). (**f**) The CNP is finally placed at the midpoint.

The presence of a failure zone usually indicates that there is a narrowing in the navigable space nearby. In this work, the exact points where such narrowings are located will be denoted by CNPs (refer to Figure 2). It is worth mentioning that, in general, these CNPs should be traversable points for the robot, since they belong to the previously built map. However, precisely because of this, the followed trajectory in these points was manually chosen by the operator and, unfortunately, might not be always reproducible by automatic path planners, as they rely on optimizing some cost functions subject to criteria that may not be as flexible as the operator’s judgment [44]. Thus, to properly navigate through the CNPs, we propose to automatically define a pair of so-called ANPs associated with each CNP that will be employed as intermediary waypoints to properly reach the desired target.

It is important to note that all these elements are related to the concept of cost-maps [15], which delimit the navigation area of a robot within a built map taking into consideration the robot footprint and the presence of obstacles. In short, two main cost-maps are typically defined for every environment, namely: global and local cost-maps. The former describes the static obstacles in the map (e.g., walls, doors, furniture, etc.) that were detected during the map building procedure. This cost-map is employed by a global planner to generate an initial trajectory between the current robot position and the desired navigation target. The latter, in turn, takes into account the current surroundings of the robot (i.e., the current sensory data) to describe those objects that were not present in the initial map but they are now visible by the robot. This allows a local planner to generate a short-term trajectory that might deviate from the global plan with the aim of negotiating these unexpected obstacles and successfully reaching the destination. Although other cost-maps can also be defined to address different aspects such as customized restricted areas, social constraints [45], or robot behavior control, they can be seamlessly merged into a final cost-map that can be employed in the same way as those used here. However, an in-depth study about them falls beyond the scope of this paper.

In the following sections, we thoroughly describe the algorithms developed to, first, automatically find both the failure areas and the CNPs, and, then, to generate the ANPs.

### 3.1. Automatic Detection of Critical Navigation Points (CNPs)

Our proposal initially takes two elements as input:

- The 2D map that was built during the deployment of the robot at home, typically by means of aligning the sensor readings (e.g., from laser scanners) that were recorded during the first exploration of the robot’s operation space.
- The list of nodes or destination points at the different rooms in the house. This is normally performed manually by the operator, selecting those rooms where the robot is expected to carry out some task, while avoiding others like bathrooms where the robot presence may not be welcomed by the end user.

From these input data, our method generates all possible trajectories between all the existing nodes and, subsequently, commands the robot to navigate autonomously following every trajectory while detecting either collisions, grazes or areas of difficult access, ultimately leading to the identification of the failure zones. It is important to notice that a successful navigation between two nodes does not ensure the same result for the opposite trajectory, hence being also necessary to evaluate the return path navigability.

Every time a failure zone is found, our proposal automatically generates a CNP at the most probable location that could be responsible for the problem, as described in Algorithm 1. On most occasions, these CNPs correspond to narrow areas such as doorsteps, corridors, corners, etc., To generate them, a window covering the failure zone is taken from the global cost-map and inspected in search of the edges of the navigable space (see Algorithm 1, steps 1 and 2). These edges are formed by the so-called lethal points, which represent the locations in the map where collision with an obstacle is to be expected if the robot’s center falls within them (refer to Figure 2). Then, for each pair of found edges ($e_{1}$ and $e_{2}$), the Euclidean distance between all the points in $e_{1}$ and every point in $e_{2}$ are computed, finding those points ($p_{1}$ and $p_{2}$) that yield the shortest distance (see Algorithm 1, steps 3–9), which corresponds to the narrowest navigable area of the map within the failure zone. Notice that, since this search is run within a small window around the failure zone, there is no need for a more elaborated procedure to find the minimum distance, as the improvement in efficiency would not be really significant. Finally, a CNP is set in the middle of such narrow location (see Algorithm 1, step 10).

**Algorithm 1:** Determining the critical points (CNPs)

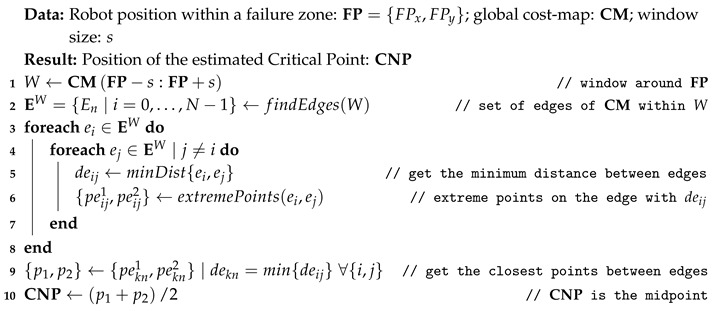



### 3.2. Generation of Auxiliary Navigation Points (ANPs)

**Figure 3:**
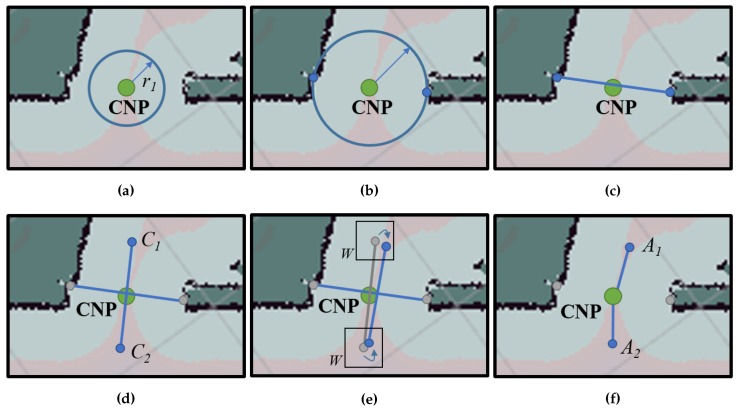
Generation of auxiliary navigation points (ANPs). (**a**) A growing circumference is created centered at the CNP until (**b**) two points of the cost-map touches it, (**c**) so that they are approximately aligned with the CNP. (**d**) A perpendicular line is generated and two candidates ($\left\{C_{1},C_{2}\right\}$) for the ANPs are placed. (**e**) The positions of the candidate points are refined according to the cost-map, (**f**) yielding the final ANPs locations ($\left\{A_{1},A_{2}\right\}$).

As mentioned before, once the CNPs have been identified, two ANPs ($\left\{A_{1},A_{2}\right\}$) associated with each CNP are automatically generated (refer to Algorithm 2), and subsequently employed as mandatory navigation waypoints when the robot trajectory passes through the problematic area. These two points, once properly placed, guides the robot to traverse the area following the optimal path in terms of navigation cost. An illustration of the process described here can be seen in Figure 3.

In order to find the ANPs, we first identify those points that delimit the involved narrow zone by circumscribing the largest possible circumference in the global map centered at the CNP.

**Algorithm 2:** Determining the auxiliary navigation points (ANPs) from the critical points (CNPs).

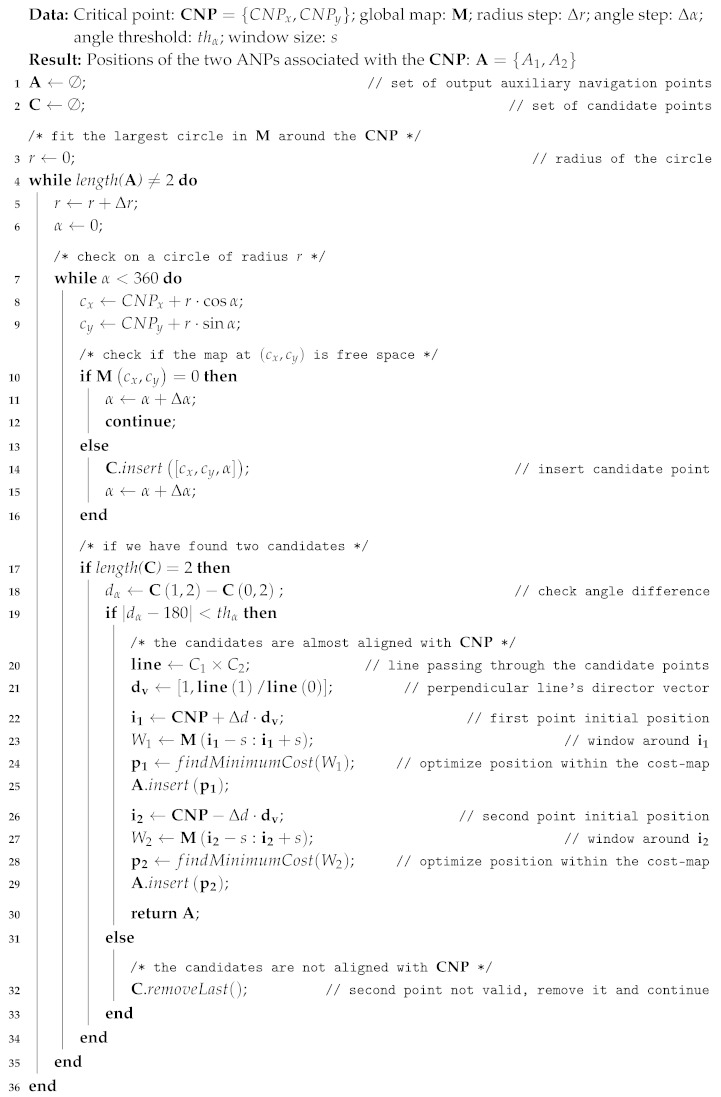



This is accomplished by generating a growing circumference while searching for obstacles that are in contact with the circumference (steps 3–36). For that, all the circumference points are checked until two candidate points ($\left\{C_{1},C_{2}\right\}$) in the cost-map are found for the same radius (step 17). Then, we check whether or not they are aligned with the CNP, allowing for a certain small tolerance (step 19). In case the condition is not fulfilled, the last candidate point is discarded and the search process is resumed. On the contrary, if they are aligned, the perpendicular line passing through them is computed (steps 20 and 21) and two tentative ANPs are generated, at a certain arbitrary-defined distance from the CNP.

Finally, we now make use again of the cost-map to refine the location of the ANPs, since we aim to locate them at the optimal position so that the robot navigation is as effortless as possible. Thus, we search for the minimum cost within a window around each provisional ANP position to eventually place them there (steps 22–25 and 26–29). All this process is performed on-the-fly during robot navigation and takes less than one second to generate a CNP and its associated ANPs.

Once all this procedure has finished, the trajectory $A_{1}⟶$ CNP $⟶A_{2}$ (or vice versa, if the navigation is the other way around) is defined as the path to follow by the robot in order to overcome the navigation problem found at the CNP.

## 4. Experimental Setup

To evaluate the proposed navigation assistant and its impact on realistic deployments of robots at homes, we introduce in this section the set of maps gathered from real houses to be considered for experimentation (see Section 4.1). Furthermore, different robot shapes will be taken into account, corresponding to real robots that have been tested and deployed in different research projects or that are commercially available with the main focus to assist the user at home (Section 4.2). Finally, we briefly describe the selected configuration of the navigation algorithms and highlight the values of the most important navigation parameters (Section 4.3).

### 4.1. Laser-Based Maps of Real Houses

**Figure 4:**
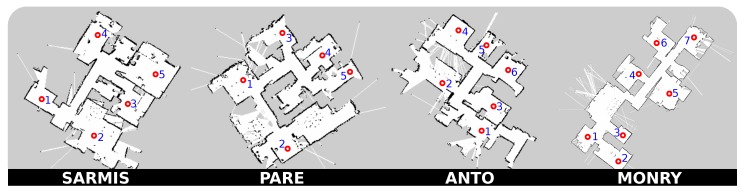
Floor-plans of the four houses where navigation will be tested. These geometric maps are built by deploying a mobile robot in the house and teleoperating it while running a SLAM algorithm based on 2D laser range measurements.

To evaluate the navigation performance of the robots in multiple realistic environments, we will consider four different maps corresponding to real houses (see Figure 4). It is to be stressed that all these maps were generated with a robot being deployed in a real house, using a 2D laser rangefinder and SLAM algorithms. Concretely, to generate those 2D geometric maps we used GMapping [46], a Rao–Blackwellized particle filter approach based on occupancy grids. This algorithm is fed with the data streams of odometry [47] and the laser scanner range observations of a Hokuyo URG-04LX-UG01. A detailed description of each one is presented next.

- SARMIS: this map corresponds to a (9.8 × 10.7) [m] old-style house with wide rooms and a small corridor. We selected five out of the seven rooms to test navigation, as some of them have a really narrow entrance (up to 54 cm in some cases), which can be potentially not reachable by most robots.
- PARE: this map represents a (10.15 × 9.43) [m] flat-style house with clear square shape and absence of a long, dominant corridor. Discarding one of the bathrooms for the aforementioned reasons, we selected a total of six rooms for testing purposes. The presence of dots in the maps represents a high density of furniture (e.g., tables and chairs) which will indeed make navigation more challenging.
- ANTO: this map has been built from a (12.33 × 8.9) [m] house with a small central corridor that connects the six rooms that compose it. Though challenging, we consider all the rooms of the house for testing navigation.
- MONRY: this map corresponds to a (17.6 × 7.5) [m] elongated shape flat with a dominant, long and narrow corridor where most of the rooms are connected to. It is composed of a total of eight rooms from which we selected seven, discarding again a bathroom.

The fact that the first three of the four chosen datasets belong to the Robot@Home dataset [48] is due to the lack of publicly available databases that contain occupancy grids of real houses, being usually focused on labs and offices instead.

### 4.2. Robotics Platforms

**Figure 5:**
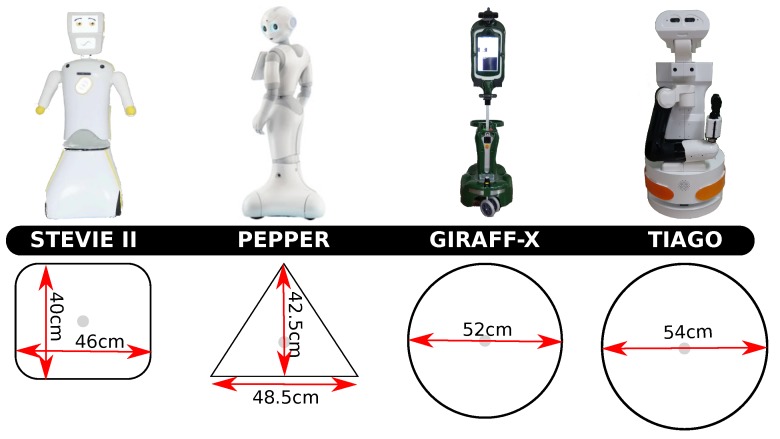
Pictures of the four robots considered in the experimental section together with their respective footprints. The robots have been ordered according to the dimensions of their footprints from left (smallest) to right (biggest).

In order to evaluate different robot shapes and dimensions, we have selected the four different robot footprints shown in Figure 5. All of them correspond to companion robots designed to assist the user at home, which either have been developed as part of EU projects or are commercially available. Notice that from the range of assistant robots in the literature, we have selected the four that maximizes the diversity of shape and dimensions, while discarding those too big to be able to navigate the selected house environments (as it is the case of Care-o-Bot, PR-2 or Scitos-G5, for example). Next, we provide a short description of the selected robots, focusing on their footprints as the parameter with a larger impact on their navigation capabilities.

- Stevie II [49]: this is the follow-up version of the robot Stevie, who served as a proof of concept of the fact that a socially assistive robot can be deployed in long-term care environments to help seniors and people living with disabilities. Stevie II, which is used within the EPIC EU project, has been built on the project successes and also embodies significant technological upgrades and advanced AI capabilities. It presents a small rectangular footprint (the smallest one in this study), and a height of 130 cm approximately.
- Pepper [50]: developed by Aldebaran Robotics, it is a mobile robot featuring a three-wheeled platform with a triangular footprint. The robot is about 120 cm high, weighs 28 kg and it is equipped with cameras, microphones, speech recognition and social intelligence. It has been designed with the purpose of acting as a companion for the elderly, a teacher of schoolchildren and an assistant in retail shops, among other uses.
- Giraff-X [16]: an active companion robot designed for the assistance of elderly people in their daily life. The Giraff robot, which has been designed and evolved through multiple EU projects: Excite [51], Giraff-Plus [52] and MoveCare [9], is a robotic platform endowed with autonomous navigation capabilities, user interaction, visual object detection, and semantic mapping among its most important skills. Its footprint can be approximated by a circle and contains two motors and two caster wheels, as well as an adjustable height reaching up to 170 cm.
- TIAGO [53]: the versatile, modular robotic platform from Pal Robotics used along different EU projects as EnrichMe [54] or GrowMeUp [55]. It presents the biggest footprint among the compared robots, and allows an adjustable height between 110 and 145 cm.

### 4.3. Navigation Parameters

The configuration and tuning of the navigation parameters is not a trivial task, becoming even more complicated when the environment where the robot must navigate is a house (i.e., usually a narrow and cluttered environment). This task is, however, of capital importance in order to achieve satisfactory results, being necessary the operation of qualified personnel and, generally, several hours of trial and test series. In this section, we describe the configuration that will be used during the experiments regardless of the map or the robot shape. That is, we tuned the navigation parameters to achieve the best performance in our laboratory (see for example the tuning guide [56]), and then applied it to all the test cases. Naturally, fine-tuning the parameters according to the specific robot shape and the environment characteristics could slightly improve the results, but overall the conclusions would remain.

We estimate the robot pose using AMCL [57], the ROS implementation of the popular Monte-Carlo Localization (MCL) algorithm proposed by Fox et al. [58]. This algorithm takes as input the laser scanner range measurements, odometry data, and a standard 2D occupancy grid map. AMCL maintains internally the motion and measurement models and uses them to iteratively resample the position and orientation of a pool of particles representing the belief on the robot pose. For navigation, we employ the commonly used move_base stack [59], setting NavfnROS as the global planner and the DWA algorithm as local planner. We configure the 2D cost-maps to account for the static layer (i.e., the geometric static map), the obstacle layer (taking only into consideration the measurements of the 2D laser rangefinder for the simulated experiments, and also accounting for the 3D measurements of the RGB-D cameras placed on the Giraff-X robot during the real experiment), and the inflation layer. The latter controls the cost of traversing the space near the obstacles, either static or dynamic. For this, we take into consideration the different robot shapes, updating the robot footprint accordingly and ensuring that the inflation_radius parameter is always greater than the inscribed radius of the robot footprint. An important parameter to be remarked is the resolution of the cost-maps. Given the robot dimensions and the narrow areas usually present in the houses, it is recommended to increase the cost-map resolution up to 1 cm per pixel, in order to ensure that the planners have a range large enough to test different trajectories.

## 5. Experimental Results

This section evaluates the capabilities of our navigation assistant when deploying a robot in a real environment, assessing the navigability of the robot when following the paths generated by our proposal. For this, we first present in Section 5.1 the results of a set of simulated experiments where we consider all the house environments and robot shapes introduced in Section 4, and, then, addresses in Section 5.2 a real experiment with the Giraff-X assistive robot navigating in a real house, aiming to assess the performance in more realistic scenarios.

The simulated experiments have been performed in Stage [60], which is a fairly complete, yet simple, ROS-compatible simulator that provides computationally efficient models of lots of devices, sensors and robot shapes. Thus, it is able to reproduce odometry readings for the different robot shapes as well as laser scanner noisy measurements.

### 5.1. Experiment #1: Simulated Robots in Real Maps

In our experiments, we employ the navigation success rate (NSR) as the metric for the evaluation of the improvement obtained when considering our proposal. In this work, the NSR represents the number of successful autonomous navigations (without user/technician intervention) between two nodes in the house, divided by the total number of navigation paths available in the house. We have considered the going and return paths separately, as they may have different results. Thus, as an example, a house with four nodes leads to 12 possible paths: 1⟷2, 1⟷3, 1⟷4, 2⟷3, 2⟷4 and 3⟷4, where a 67% NSR would correspond to eight successfully navigation attempts out of twelve.

**Figure 6:**
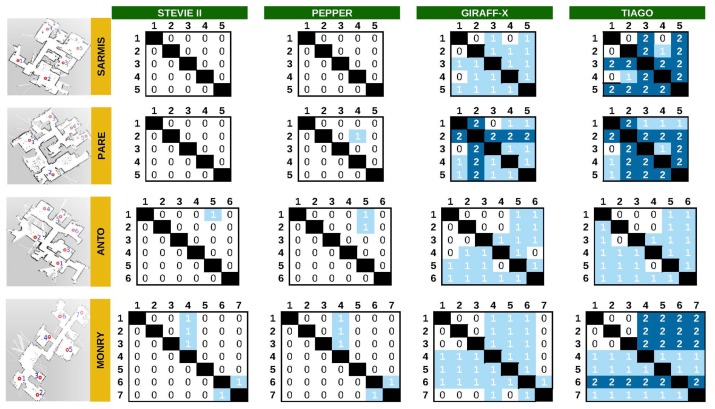
Navigation results for the different robots and test environments. The results are labeled as 0: no CNP detected, navigation without error, 1: one or multiple CNPs detected, navigation successful only after considering the ANPs, or 2: one or multiple CNPs detected but navigation failed even after considering the ANPs.

Table 1 summarizes the results for the different environments and robot shapes, with and without the proposed navigation assistant. We consider as success those navigation attempts where the robot is able to reach the target without problems, and failure when there is either a collision (even if it is only grazing a wall or a door frame) or when the robot gets stuck, not being able to find a valid trajectory even after executing some typical recovery behaviors (e.g., re-localization, small movements to elude local minima, etc.). A more detailed evaluation of the experimental results is presented in Figure 6, where the navigation outcome for each possible path is depicted. In the tables shown in this figure, the cell at $\left(i,j\right)$ represents the outcome of the navigation from node *i* to node *j*. The value 0 stands for a successful navigation without any CNP detected. In turn, the value 1 indicates that one or multiple CNPs were detected and the navigation succeeded after using the ANPs associated with them, proving the usefulness of our approach. Finally, a value of 2 represents unsuccessful navigation even with the generation of the ANPs.

**Figure 7:**
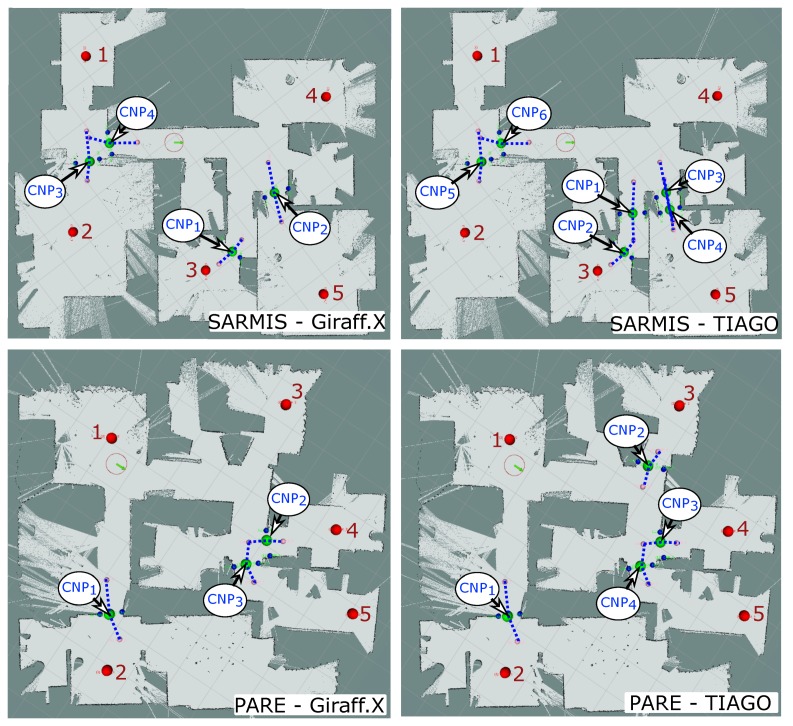

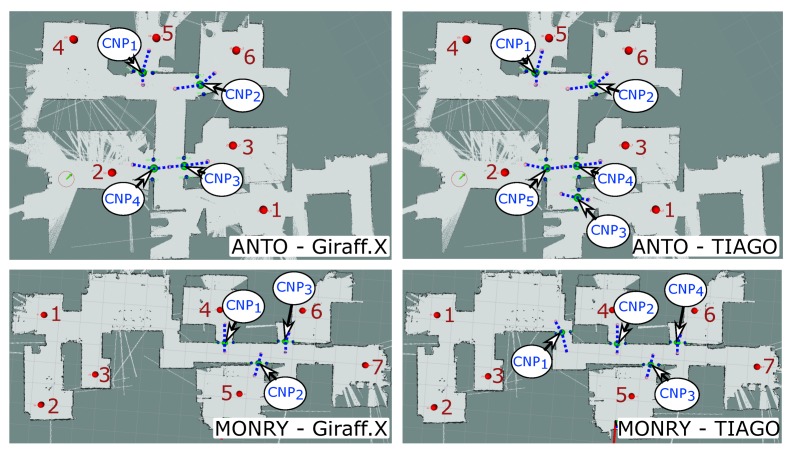
Illustration of how different robot shapes lead to the detection of different CNPs in the environment. Nodes are displayed as red dots and numbered according to the order used in the experiments, CNPs have been explicitly marked and numbered according to the order they are created during the experiments, and ANPs are marked with a blue-dash line that joins the two ANP belonging to the same CNP.

Multiple conclusions can be extracted from these results:

- The proposed navigation assistant equals or improves the navigation success rate for all the robot shapes and tested scenarios, reaching improvements of up to 80% (e.g., see Giraff.X results for the SARMIS map, where all the issues were fixed). Nonetheless, when the robot dimensions are too big for a given environment, the improvements are more humble (e.g., see TIAGO results for the SARMIS, PARE or MONRY maps in the last column in Figure 6). These unreachable locations are caused, in most cases, by the reactive planner being unable to calculate a safe navigation path between the ANPs due to the proximity of obstacles, leading to aborting the navigation. Even in these cases, though, our system is capable of increasing the number of successful navigations.
- To ensure an error-free operation of the robot at home, it is recommended to keep a dimension-security-margin of at least 12 cm. That is, the most restrictive robot dimension should be at least 12 cm smaller than the narrowest area in the environment (i.e., doors, corridors, etc.). Both Pepper and Stevie II present little navigation problems given their small footprints in comparison with the house maps used in the experiments (where the narrowest areas corresponded to doors with a size of between 60 cm and 70 cm). Only in a few scenarios, one or two paths are not successfully followed without help (but they are fixed by our assistant), while for the Giraff-X or TIAGO, the navigation failures rise considerably. The reason behind this dimension-security-margin are the multiple sources of error that play a role in the autonomous navigation of the robot, namely: the error related to the laser measurements when sensing the environment, the resolution of the grid-map used to represent the occupancy map (being advisable to increase the resolution as much as possible according to the computational power of the robot), and last but not least, the errors due to the path planning of the robot, where even if a valid global path can be found (i.e., theoretically the robot should be able to pass), its navigation leads to small deviations that can be problematic on too narrow areas.
- When the robot size is close to that of the narrow areas of the environment, a high failure rate in the autonomous navigation is to be expected. It is in these cases where finding the CNPs, either by manually setting them based on expert knowledge or by employing our proposed navigation assistant, becomes mandatory. By forcing the robot to cross those problematic areas in a specific way (i.e., by means of setting the ANPs), high success rates can still be achieved in most scenarios as can be noticed from the results of the Giraff.X robot, where most of the wrong navigations can be solved.
- From the multiple experiments and several navigation attempts, we have learned that the most controversial areas leading to faulty navigation are those involving a narrow area and a circular-like robot trajectory to transverse them. That is, the success rate is usually much higher when the robot is able to plan a path to cross a narrow area (i.e., a doorstep) employing an approximately straight trajectory. See for example the high error rate for Giraff.X or TIAGO when navigating to/from rooms 4, 5 or 6 in the MONRY map, which involves a 90${}^{\circ }$ turning to either enter or leave the room. In contrast, for the same map and robots, room 7 (which has the same doorstep size) does not present problems for the navigation (refer to Figure 7 (bottom), where it can be seen how the entrance to rooms 4, 5 and 6 have been marked as CNPs while room 7 has not).
  Interestingly, the typical distribution of rooms in a house promotes paths that heavily turn on the doorsteps and corners to reach the different destinations, becoming, therefore, challenging for big sized robots. These scenarios represent the core of our proposal, producing ANPs to enforce the robot to turn in place and traverse the CNPs following paths as straight as possible, hence solving most of the problematic navigations.
- Regarding the automatic detection and characterization of the critical points in the environment, our proposal has demonstrated to be robust and versatile, successfully locating the set of CNPs and ANPs in most situations (see Figure 7). Yet, like any other algorithm, it is not exempt from failures, being advisable the supervision of a technician to ensure maximum coverage of the navigation area within the house during the robot deployment phase, especially with large robots and/or small environments. In any case, our navigation assistant provides a suitable initial proposal of critical zones that, if needed, can be further fine-tuned by a technician or a robotic practitioner.

### 5.2. Experiment #2: Real Robot in a Real Environment

To reaffirm the results obtained during the simulation experiment, we have conducted a real experiment consisting of the real deployment of the Giraff.X robot at the MONRY house environment. To properly evaluate the differences with its simulation counterpart, we carried out a clean deployment of the robot, that is: (i) we manually guided the robot to create the geometric map of the house, (ii) we set the navigation goals (i.e., *nodes*), and, finally, let the navigation assistant to determine the location of the CNPs and ANPs, if needed. A peculiarity of the Giraff.X robot is the lack of bumpers to detect collisions, one of the indicators of the presence of a navigation failure point. To overcome this issue, we have made use of its interaction buttons (i.e., a green and red buttons, commonly used by the end-user to accept and reject actions or proposals from the robot), configuring the red button to notify to the navigation assistant that a collision had occurred. It is worth mentioning that if the robot base included bumpers, this process could be easily further automated and no human intervention would be required.

**Figure 8:**
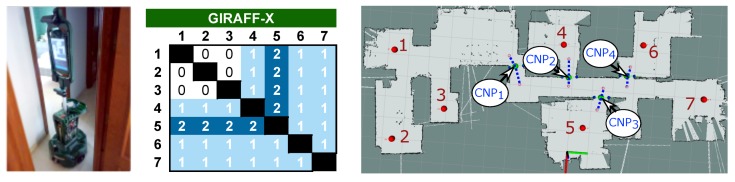
Picture of the Giraff.X robot during the real experiment (**left**) and results obtained (**middle**) for each navigation path in the environment (**right**).

The results of this experiment are detailed in Figure 8. As can be seen from the node-to-node navigation table, the success rate is, as expected, a bit worse than in simulation (refer to the table in row 4 and column 3 in Figure 6). This difference appears mainly due to the slight dissimilarities between the real conditions that might affect the navigation and the simulation of the scenario, leading to worse results in terms of navigation for the real experiment in comparison to its simulated counterpart. In this case, the navigation capability of the Giraff.X robot in this challenging environment degraded to a poor 19.05% when operating unassisted. Nevertheless, our proposed navigation assistant significantly improves its performance, rising the NSR up to 80.95%. With respect to the detection of the CNPs, the main differences with the simulation case are (i) the creation of an additional CNP (CNP${}_{1}$ in Figure 8 (right)), and (ii) the failure to navigate to/from node 5 even after the consideration of the CNP${}_{3}$ and its associated ANPs. The former corresponds to the narrow corner at the edge of the corridor connecting nodes 1–3 with 4–7, which, in some occasions, made the robot to get stuck, so an additional CNP had to be automatically created to solve this issue. The latter is related to the location of the ANP close to node 5. Given that the ANPs are set according to the location of minimum cost (see Section 3.2), in practice, the presence of a shelf at one side of the door entrance makes the ANP to be moved too far from the perpendicular line to the CNP, leading the robot to graze the door-frame or even to get stuck when trying to cross it. This is the type of situation that would require a fine-tuning of the ANPs position by a human supervisor. A video of the real robot operating with our navigation assistant can be seen in http://mapir.isa.uma.es/work/paper-awg.

## 6. Conclusions

This paper has presented a navigation assistant that significantly mitigates the problems that state-of-the-art mobile robots face when operating autonomously in standard houses. In practice, traversing corridors, navigating around corners or through narrow doors become problematic during autonomous navigation mainly due to inaccuracies in the robot localization, noise and errors in the sensor measurements, and unsuitable trajectories generated by widely-employed path planners, often leading to unsuccessful navigation and unreachable areas in the environment. In this scenario, the detection of such problematic areas (if addressed) is often left to a technician who has to decide the particular trajectory the robot must follow to overcome the navigation problem. This is typically performed by hand, following their intuition or after performing some navigation tests.

Our proposed navigation assistant detects, without any human intervention, points of difficult navigation, which are denoted by CNPs. From them, it automatically generates a set of ANPs that effectively modifies the robot trajectory so that such problematic areas can be traversed effortlessly. This approach leads to a significant increase in the NSR, i.e., the ratio between the number of completed navigations and all possible navigations between the nodes in the map. Our system has been validated through a series of simulated experiments based on maps built from real houses, and the footprints of four state-of-the-art mobile robots employed in recent and current EU projects, namely: Stevie II, Pepper, Giraff.X and TIAGO. Another experiment involving the Giraff.X mobile robot [16] has also been presented, this time navigating in a real environment.

The results show that our approach is especially useful for robots with large bases (larger than 50 cm). As an example, for the Giraff.X robot, with an almost circular footprint with a diameter of 52 cm, our navigation assistant increases the NSR from 0.2 up to 1.0 in the SARMIS map (i.e., all navigations are fixed), although more humble improvements are achieved in other scenarios: from 0.1 to 0.6 or from 0.2 to 0.3 for the TIAGO robot, also in the SARMIS map. In any case, the impact of generating the ANPs as proposed in this work is proven to be positive for all scenarios. In the experiment with the real robot, slight differences appear in comparison to its simulated counterpart regarding the generation of the CNPs and the ANPs, mostly due to inaccuracies in sensory data and robot localization. Nevertheless, even in this case, our system increases the NSR from 0.2 to 0.8.

In the future, we plan to adapt the generation of the ANPs so that they can be inspected for long-term usefulness. This relies on the fact that some ANPs might be created due to a temporary change in the map, and their presence is no longer required after the change has been removed. This extension can be addressed by checking that, through time, the ANPs are still useful and normal navigation keeps being cumbersome at that point, being removed otherwise. This would definitely enhance the usefulness of our approach since it would provide an automatic adaptation of the navigation trajectories for dynamic environments and would avoid the need of explicitly triggering new exploration stages from time to time. Another future work involves the fusion of different ANPs associated with different but nearby problematic zones, which might have been created very close to each other. In this situation, they can be merged so that only an intermediary navigation waypoint is kept, hence reducing potentially unnecessary robot movements. Finally, the development of an autonomous procedure for the exploration and annotation of the environment would allow our system to operate without any human intervention from the very beginning of the robot deployment at home. This would need, though, some posterior manual refinement to remove areas that the user does not want to keep as potential navigation goals.

## Figures and Tables

**Table 1:** Results of the simulated experiment for the different robots and house environments. For each combination, the navigation success rate (NSR) when employing standard navigation algorithms based on the widely employed ROS move_base stack (MB), and when using the proposed navigation assistant are depicted. Average values are also included. Values in bold represent the best NSR for each combination.

		Robotic Platform							
		Stevie II		Pepper		Giraff-X		TIAGO	
		MB	NA	MB	NA	MB	NA	MB	NA
**House Env.**	**Sarmis**	**1.0**	**1.0**	**1.0**	**1.0**	0.2	**1.0**	0.2	**0.3**
	**Pare**	**1.0**	**1.0**	0.95	**1.0**	0.1	**0.6**	0.05	**0.35**
	**Anto**	0.97	**1.0**	0.93	**1.0**	0.37	**1.0**	0.23	**1.0**
	**Monry**	0.88	**1.0**	0.88	**1.0**	0.36	**1.0**	0.14	**0.57**
	*average*	0.96	**1.0**	0.94	**1.0**	0.26	**0.9**	0.16	**0.56**
